# Transcriptional Analysis of the Early Ripening of ‘Kyoho’ Grape in Response to the Treatment of Riboflavin

**DOI:** 10.3390/genes10070514

**Published:** 2019-07-06

**Authors:** Zhen-Guang Wang, Li-Li Guo, Xiao-Ru Ji, Yi-He Yu, Guo-Hai Zhang, Da-Long Guo

**Affiliations:** 1 College of Forestry, Henan University of Science and Technology, Luoyang 471023, Henan Province, China; 2 Henan Engineering Technology Research Center of Quality Regulation and Controlling of Horticultural Plants, Luoyang 471023, Henan Province, China; 3 College of Agriculture, Henan University of Science and Technology, Luoyang 471023, Henan Province, China

**Keywords:** early ripening, grape, Kyoho, riboflavin, RNA-seq

## Abstract

Previous study has demonstrated that the riboflavin treatment promoted the early ripening of the ‘Kyoho’ grape berry. However, the molecular mechanism causing this was unclear. In order to reveal the regulation mechanism of riboflavin treatment on grape berry development and ripening, the different berry developmental stages of the ‘Kyoho’ berry treated with 0.5 mmol/L of riboflavin was sampled for transcriptome profiling. RNA-seq revealed that 1526 and 430 genes were up-regulated and down-regulated, respectively, for the comparisons of the treatment to the control. TCseq analysis showed that the expression patterns of most of the genes were similar between the treatment and the control, except for some genes that were related to the chlorophyll metabolism, photosynthesis–antenna proteins, and photosynthesis, which were revealed by the enrichment analysis of Gene Ontology (GO) and Kyoto Encyclopedia of Genes and Genomes (KEGG). The differentially expressed genes and weighted gene co-expression network analysis (WGCNA) analysis identified some significantly differentially expressed genes and some hub genes, including up-regulation of the photosynthesis-related *ELIP1* and growth and development-related *GDSL*; and down-regulation of the oxidative stress-related *ATHSP22* and berry softening-related *XTH32* and *GH9B15*. The results suggested that the riboflavin treatment resulted in the variations of the expression levels of these genes, and then led to the early ripening of the ‘Kyoho’ berry.

## 1. Introduction

Grape (*Vitis vinifera* L.) is one of the most important cultivated fruit crops in the world. Grape berries are mainly used for fresh consuming (30%), wine making (68%), raisins, and juice (2%) [1]. Berry ripening is a complex, highly synergistic growth and development process [2]. Some physiological and biochemical metabolism processes are markedly altered during the berry-ripening process, such as the production of fragrance, firmness of texture, color changes, and sugar metabolism [3], which affect the quality of the fruit to some extent.

‘Fengzao’ is an early ripening bud mutant of ‘Kyoho’ [4] that matures 30 days (d) earlier than ‘Kyoho’. Comparative RNA-seq analysis of these two grape varieties by Guo et al. [2] showed that the significantly differentially expressed genes were related to reactive oxygen species (ROS), and the expression of the *SOD* (superoxidase dismutase) gene in ‘Fengzao’ is significantly lower than that in ‘Kyoho’. Furthermore, the differences of ROS metabolism during the berries’ development periods between the two varieties were revealed [5].

ROS play an important role in signaling under biotic and abiotic stresses [6,7,8]. ROS as a signaling molecule was related to fruit growth and ripening in peach [9]. ROS was produced along the fruit development, but it also has high oxidation capacity and high toxicity for the fruit at the same time. ROS often damages proteins, lipids, carbohydrates, and DNA, causing severe cell membrane damage and inducing programmed cell death [10,11]. The ROS production increased during tomato ripening [12] and the decrease of the ROS content delayed the senescence of tomato [13]. In addition, ROS accumulation resulted in the ripening and senescence of peach fruit [14]. The ROS promoted the degradation of cellular wall polysaccharides and resulted in the fruit softening [15]. In brief, the accumulation of ROS is related to fruit ripening [16].

SODs are the first line to defend against the oxyradical mediated damage [17]. Oxyradical produced from riboflavin by autooxidation under light conditions could be disproportionated by SODs. Riboflavin was sprayed on ‘Kyoho’ young berries as the exogenous reactive oxygen species, which accelerated the ripening of ‘Kyoho’ 16 d earlier than the control [18].

The above results indicated that the endogenous ROS play a role in the grape berry ripening. To investigate the transcriptional mechanisms of the early ripening of the berries treated with riboflavin, RNA-seq was used to characterize the differentially expressed genes related to riboflavin treatment.

## 2. Materials and Methods

### 2.1. Experimental Plant Materials

Five-year-old ‘Kyoho’ grapes were cultivated with hedgerow trellis in the fields of Henan University of Science and Technology. The concentration gradient of riboflavin was set as 0.1 mmol/L, 0.5 mmol/L, and 1 mmol/L, which were dissolved with 1 mol/L of NaOH and mixed with Silwet-77 surfactant (0.03%, *v/v*). Riboflavin was sprayed evenly on the clusters of ‘Kyoho’ twice; the first spraying was at 40 days post-anthesis, and the second was at 50 days post-anthesis in 2016. Distilled water (including 0.03% Silwet-77 surfactant with final concentration and equivalent NaOH solution) was sprayed on berries as the control. Each treatment consisted of three repetitions with five trees per repetition. The specific treatment and sampling information are shown in Table 1. The berries without diseases and insect pests were sampled with 30 berries per repetition. Then, the collected berries were immediately wrapped in tin foil, frozen in liquid nitrogen, and stored at −80 °C until use.

### 2.2. Total RNA Extraction, Library Construction, and RNA-seq

Total RNA was extracted from berries using the TIANGEN kit manufactured by TIANGEN biochemical technology (Beijing) Co. LtD. According to the instructions of the kit, the RNA of the treatment (0.5 mmol/L riboflavin) and the control (water) were extracted from the berries of each stage. The total RNA was treated with RNase-free DNase I (Takara, Tokyo, Japan) at 37 °C for 15 min to remove contaminating DNA. The concentration of RNA was checked using Qubit RNA Assay Kit in Qubit 2.0 Flurometer (Life Technologies, CA, USA). The library was constructed with five steps: (1) First, 1 μg of total RNA of per sample was used to purify mRNA with poly-T oligo-attached magnetic beads. (2) The mRNA was randomly interrupted. (3) The cDNA was synthesized using random hexamers and purified with AMPure XP beads. (4) The purified double-stranded cDNA was subjected to end repair. (5) The cDNA library was obtained by PCR enrichment. The non-strand specific libraries were used for Illumina Hiseq 2500 paired-end sequencing at the Beijing Biomarker Technologies Co. Ltd (Beijing, China).

### 2.3. De Novo Assembly of Reads from RNA-seq and Functional Annotation

The raw data of RNA-seq reads were purified by trimming the adaptor sequences, duplicating sequences, reads with poly-N, and low-quality reads. The clean reads were used for sequence assembly using the trinity de novo assembly program with default parameters [19]. The sequences reads were mapped to a grape reference genome (ftp://ftp.ensemblgenomes.org/pub/plants/ release-32/fasta/vitis_ vinifera/) for further analysis using TopHat v2.0.9 [20] and Bowtie [21]. To functionally annotate genes, the unique sequences were annotated against public databases, including the National Center for Biotechnology Information non-redundant protein (Nr) (http://www.ncbi.nlm.nih.gov/) and Swiss-Prot (http://www.expasy.ch/sprot/), Gene Ontology (GO) [22], and Kyoto Encyclopedia of Genes and Genomes (KEGG) [23] databases.

The raw reads have been submitted to National Center for Biotechnology Information (NCBI)under the BioProject accession number of PRJNA528145 and SRA (Sequence Read Archive) accession number: SRX5546585- SRX5546604.

### 2.4. Cluster Analysis

The read numbers mapped to each gene were counted using HTSeq v0.6.1 [24]. Raw counts of the genes were normalized to the fragments per kilobase of transcript per million mapped reads (FPKM). The average FPKM value of each gene was calculated and transformed to two as the log base. The expression patterns of all the genes both in the treatment and the control were characterized by TCseq [25].

### 2.5. Differential Gene Expression Analysis

The FPKM value was employed to quantify the gene expression levels. The DEseq [26] was used to identify differentially expressed genes (DEGs) from RNA-seq data. The thresholds for the significance test were set as a false discovery rate (FDR) ≤0.01 and |Fold Change| ≥2 [27]. The enrichment analysis of GO and KEGG were done using ClusterProfiler [28].

### 2.6. Weighted Gene Co-Expression Network Analysis (WGCNA)

To identify the relevant gene modules and investigate the hub genes within the modules, weighted gene co-expression network analysis (WGCNA) was carried out with the R package [29]. The eigengene value of each module was calculated to test the correlation with each gene sample [30]. Before creating the network model, the distribution of the entire dataset of all gene-filtered and quality-controlled transcripts was performed. The hub genes from the model were screened by calculating the connectivity degree of each gene with Cytoscape 3.6.1 [31].

### 2.7. Quantitative Real-Time PCR

Six DEGs and two hub genes that related to berries ripening were selected for quantitative real-time PCR analysis. cDNAs were reverse-transcribed from total RNAs using the HiScript 1st Strand cDNA Synthesis Kit (Vazyme biotech Co., Ltd. Nanjing, China). Reactions were performed as follows: 25 °C for 5 min, 52 °C for 15 min, and 85 °C for 5 min. The primers of all eight genes were designed using Primer Premier 5.0 software, and all of the primers are listed in Supplemental Table S1. The ubiquitin gene was used as the reference gene. Three biological replicates were conducted for each sample. The qRT-PCR was performed using TransStart Top Green qPCR SuperMix kit (TRANSGEN, Beijing China). Reactions were performed as 40 cycles of 94 °C for 30 s, 94 °C for 5 s, and 60 °C for 30 s. The relative expression levels of the target genes were calculated using the 2^−△△CT^ approach [32].

### 2.8. Statistical Analysis

Statistical analysis was performed using SPSS statistic 21.0 software (IBM, Armonk, NY, USA). One-way analysis of variance (*p* ≤ 0.05) was conducted. Data are means ± SE from three independent biological replicates.

## 3. Results

### 3.1. Qualitative Evaluation of RNA-seq Data

The raw data of RNA sequencing reads were processed by trimming the adaptor sequences, duplicating sequences, reads with poly-N, and low-quality reads. Approximately 30.8 million clean reads and 29.08 million clean reads were obtained for the control and the treatment on average, respectively. The Q_30_ values were above 94% on average. The average GC percentages were higher than 47% (Supplemental Table S2). The clean reads were aligned to the grape reference genome (ftp://ftp.ensemblgenomes.org/pub/plants/release-32/fasta/vitis_vinifera/). A total of 30,694 genes were predicted for the grape genome, while 20,462 genes were detected as expressed in this study, and 18,844 genes could be annotated based on the Uniprot.

**Figure 1:**
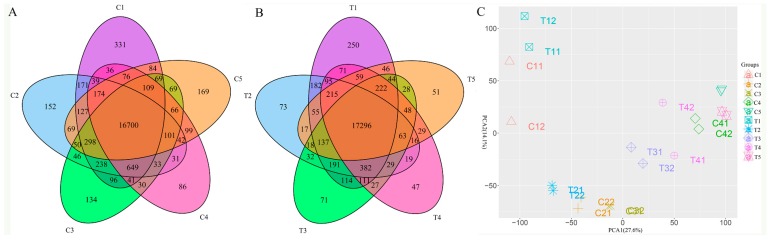
The number of expressed genes at different sampling stages and the demonstration of principal component analysis. (**A**) The Venn diagram showed the number of the expressed genes in the control group; (**B**) The Venn diagram showed the number of the expressed genes in the treatment group; (**C**) The principal component analysis showed the uniformity of the samples. Note: The representative meaning of the code for the groups was shown in Table 1. The same is below.

To investigate the differences among the expression of the genes, a Venn diagram was drawn with the Venn Diagram package (Figure 1). Figure 1A showed that the amounts of expressed genes from the control groups were 19,238, 18,920, 18,729, 18,312, and 18,272, respectively; the shared expressed genes in all the control groups were 16,700. Figure 1B showed that the numbers of the expressed genes in the treatment groups were 19,470, 18,820, 18,813, 18,729 and 18,339, respectively; while there were 17,296 shared genes in this group. Compared with the control, the shared genes in the treatment groups were higher than the control group on average. To assess the uniformity of the examined samples, principal components analysis (PCA) was performed (Figure 1C). The first and the second principal component explained 27.6% and 14.1% of the variance, respectively. The replicates of the control (C group) and the treatment (T group) grouped individually well; and the samples from the different groups, either from the control or from the treatment, were distinctly separated and away from one another. As a whole, the PCA showed that the repeatability of the samples was reliable.

### 3.2. Comparison of Overall Expression Patterns between the Control and the Treatment

**Figure 2:**
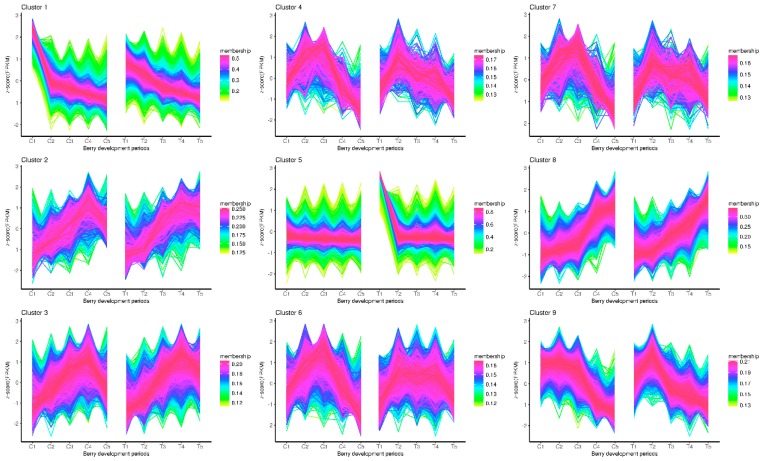
Cluster analysis of the gene expression patterns of both control and treatment across the berry development stages.

The expression patterns of the genes detected in the control and the treatment group were depicted based on the analysis of the TCseq package [25]. The division of clusters were calculated with the vegan package. As shown in Supplemental Figure S1, the highest calinski criterion value occurs at nine, suggesting that the optimal number of clusters was nine. Therefore, the total gene expression patterns were divided into nine categories. The expression patterns of most of the clusters were very similar between the control and the treatment (Figure 2). Nevertheless, there were some interesting different patterns revealed, such as in cluster 1 and cluster 5. Cluster 1 showed that the expression of the genes was rapidly downward from the C1 to C2 stage. However, the transition of the expression from T1 to T2 stage was not as drastic as from C1 to C2. Cluster 5 illustrated that the expression pattern was steady at all times from the C1 to C5 stages. While there was a distinct peak at the T1 stage, it then fell drastically in the following stages, which was obviously different from C1.

**Figure 3:**
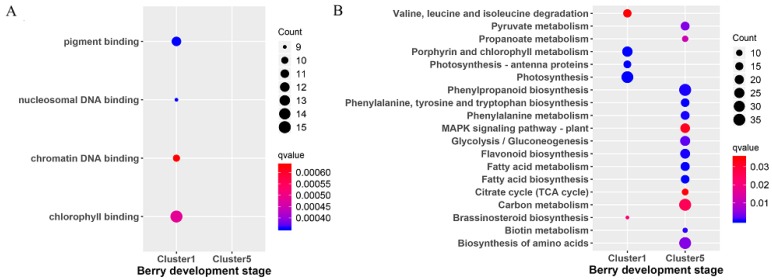
Scattergram of both Gene Ontology (GO) enrichment analysis and Kyoto Encyclopedia of Genes and Genomes (KEGG) pathways analysis. (**A**) GO enrichment analysis. The X-axis indicates the berry development stages; the Y-axis indicates the GO terms. (**B**) KEGG pathway analysis. The X-axis indicates the berry development stages; the Y-axis indicates the KEGG pathway. Coloring indicates q-value, with higher values in red and lower values in blue, and the lower q-value indicates genes that are more significantly enriched. Point size indicates genes number.

To further explore the functions of the genes in both cluster 1 and cluster 5, the GO and KEGG enrichment analysis were performed (Figure 3) with ClusterProfiler. The GO enrichment analysis revealed that the top four GO terms in cluster 1 were pigment binding, nucleosomal DNA binding, chromatin DNA binding, and chlorophyll binding (Figure 3A). There were no GO enrichment results obtained for cluster 5. The KEGG pathway analysis revealed the enrichment of 19 pathways (Figure 3B). Among that, five pathways (including valine, leucine and isoleucine degradation, porphyrin and chlorophyll metabolism, photosynthesis–antenna proteins, photosynthesis, and brassinosteroid biosynthesis) were enriched in cluster 1. Another 14 pathways (phenylpropanoid biosynthesis, glycolysis/gluconeogenesis, fatty acid metabolism, fatty acid biosynthesis, etc.) were present in cluster 5.

### 3.3. Differentially Expressed Genes Analysis 

**Figure 4:**
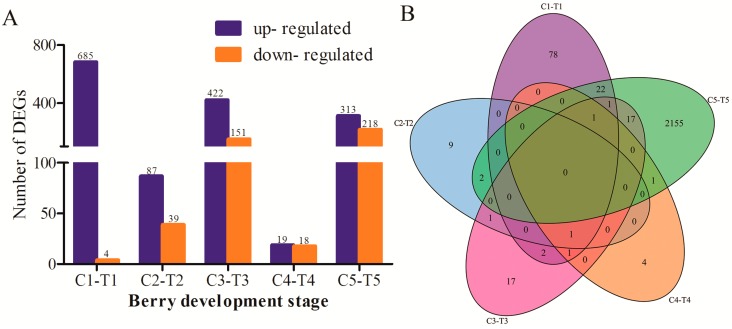
(**A**) The number of differentially expressed genes (DEGs) identified from the comparison of the control and the treatment at five stages. The X-axis indicates the berry development stages; the Y-axis shows the number of differentially expressed genes. The blue indicates up-regulation, the orange indicates down-regulation. (**B**) The Venn diagram represented the distribution of differentially expressed genes at the same stage.

To reveal the difference of expressed genes between the control and the treatment, the differentially expressed genes (DEGs) were screened at the same time point of the control and the treatment (C1-T1, C2-T2, C3-T3, C4-T4, and C5-T5). Some DEGs were shown in Table 2. A total of 1956 DEGs with significance was identified from the five stages, of which 689, 126, 573, 37, and 531 DEGs were screened from the comparison of C1-T1, C2-T2, C3-T3, C4-T4, and C5-T5, respectively (Figure 4A). From Figure 4A, it is obvious that the number of DEGs of up-regulation was higher than the number of down-regulation before the C3-T3 stage, especially for the comparison of C1-T1. However, the number of down-regulation DEGs gradually increased before the C3-T3 stage. The results revealed that the riboflavin treatment promoted some genes’ up-regulation expression. For the annotated DEGs, *GDSL* (VIT_05s0020g04840) was significantly up-regulated in T1. While in T2, *XTH32* (VIT_06s0061g00550) was significantly down-regulated. *GH9B15* (endoglucanase, VIT_02s0025g00430), *ELIP1* (VIT_05s0020g04110), and *ATHSP22* (VIT_18s0089g01270) were down-regulated in T3. Figure 4B showed the distribution of differentially expressed genes. It is obvious that the common DEGs were very low; most intersections were zero. This suggested that the riboflavin treatment had different effects on the expression of genes for each development stage.

**Figure 5:**
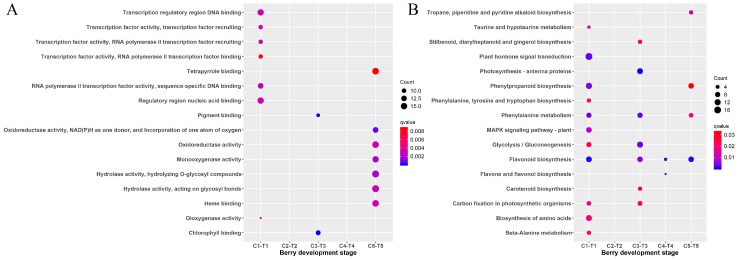
Scattergram of both GO enrichment analysis and KEGG pathways analysis of DEGs. (**A**) Top 16 pathways of GO terms enrichment among DEGs. The X-axis indicates the berry development stage; the Y-axis indicates the GO terms. (**B**) Top 16 pathways of KEGG pathway enrichment among DEGs. The X-axis indicates the berry development stage; the Y-axis indicates the KEGG pathway. Coloring indicates a q-value with higher values in red and lower values in blue, and the lower q-value indicates that the gene is more significantly enriched. Point size indicates the differentially expressed gene (DEG) number.

To identify the function of DEGs, both GO and KEGG enrichment analysis were performed. The top 15 GO terms with the most representation from the GO enrichment analysis were shown in Figure 5A. The most significant ones consisted of transcription regulatory region DNA binding and regulatory region nucleic acid binding, followed by the transcription factor activity of transcription factor recruiting; RNA polymerase II transcription factor recruiting; and RNA polymerase II transcription factor binding at the C1-T1 stage. It also consisted of pigment binding and chlorophyll binding at the C3-T3 stage. There were no results for GO enrichment analysis at the C2-T2 and C4-T4 stages.

Sixteen of the enriched KEGG pathways were shown in Figure 5B. Eleven enrichment pathways (including plant hormone signal transduction; phenylpropanoid biosynthesis; phenylalanine metabolism; and flavonoid biosynthesis, etc.) were presented in the comparison of C1-T1. There were seven KEGG enrichment pathways (including photosynthesis–antenna proteins; phenylalanine metabolism; phenylalanine metabolism, etc.) revealed in C3-T3. There were no results for KEGG enrichment analysis in C2-T2. The GO and KEGG enrichment analysis indicated that some genes associated with photosynthesis were enriched in C3-T3.

### 3.4. Weighted Gene Co-Expression Network Analysis (WGCNA)

**Figure 6:**
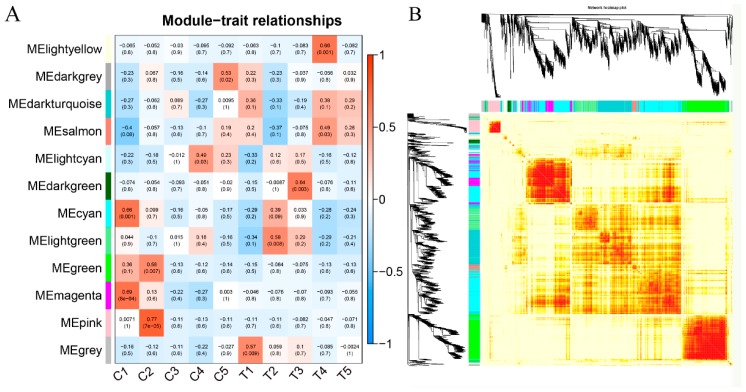
Gene modules were identified by a color name (MEcolornumber) as assigned by the weighted gene co-expression network analysis (WGCNA) package. (**A**) Heatmap correlation of berries’ differential genes of each of 12 gene modules. (**B**) Heatmap plot of topological overlap in the gene network. In the heatmap, each row and column corresponds to a gene, a light color denotes low topological overlap, and progressively darker red denotes a higher topological overlap. Darker squares along the diagonal correspond to modules. The gene dendrogram and module assignment are shown along the left and top.

To investigate the regulatory network of genes during berry development, WGCNA analysis was done to globally identify highly correlated hub genes within the highly connected gene networks based on the entire set of transcripts. Twelve modules were identified by applying the cuttreeDynamic function (Figure 6A). Each of the co-expression modules with berry development stages was identified to be associated via Pearson correlation coefficient analysis. Each cell in the Figure 6A was colored based on the statistically significance and labeled with two numbers; the upper number showed the correlation coefficient and the lower number showed the *p*-value. Figure 6A showed that the magenta, pink, light cyan, dark grey, light green, dark green, and light yellow modules were the most significantly correlated modules with the C1, C2, C4, C5, T2, T3, and T4 berry development stages, respectively.

**Figure 7:**
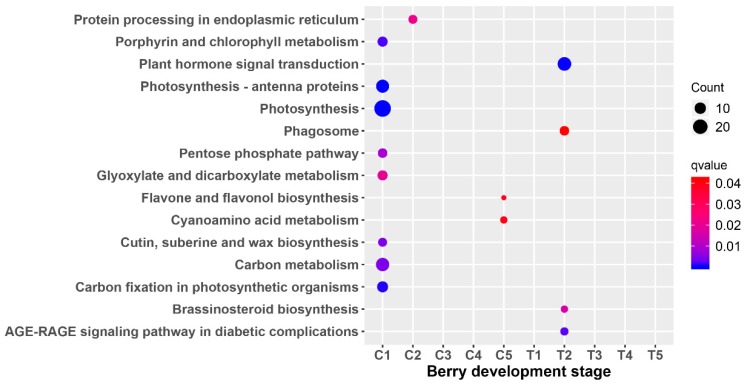
Scattergram of differential module Kyoto Encyclopedia of Genes and Genomes (KEGG) pathway analysis. The X-axis indicates the berry development stage; the Y-axis indicates the KEGG pathway. Colors indicate q values with higher values in red and lower values in blue. The lower q value indicates genes that are more significantly enriched. Point size indicates the gene number.

To further characterize the genes in the modules, both GO and KEGG enrichment analysis were performed. There was no result for the GO enrichment analysis for all the stages. The most significant ones consisted of porphyrin and chlorophyll metabolism; photosynthesis–antenna proteins; photosynthesis; the pentose phosphate pathway; glyoxylate and dicarboxylate metabolism; carbon metabolism; and carbon fixation in photosynthetic organisms at the C1 stage (Figure 7). While there were no enrichment results for T1, the most significant ones included plant hormone signal transduction; phagosome, brassinosteroid biosynthesis; and the Advanced glycation end products - receptor for advanced glycation endproducts (AGE-RAGE) signaling pathway in diabetic complications at the T2 stage. There were no results for KEGG enrichment analysis at the C2, C3, C4, T1, T3, T4, and T5 stages.

### 3.5. Reconstruction of Gene Co-Expression Network

**Figure 8:**
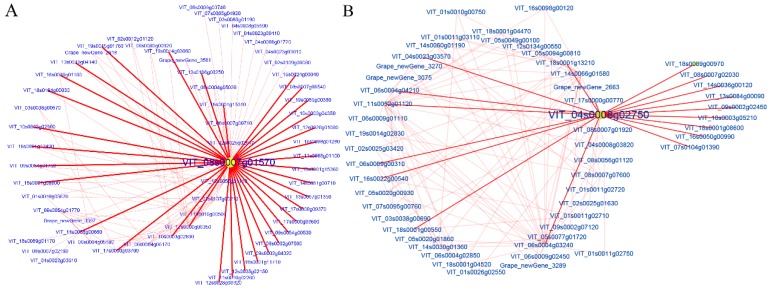
Cytoscape representation of co-expressed genes with edge weight ≥0.10. The important hub gene was noted with yellow. (**A**) The hub gene of the C1 stage and (**B**) The hub gene of the T2 stage, respectively.

For WGCNA analysis, K_ME_ values indicated the eigengene connectivity based on the assumption that hub genes are identified by sorted K_ME_ values [33]. In this study, several genes with the highest K_ME_ values in each module were selected as hub genes. The hub genes were visualized using Cytoscape software (3.6.1 version) and illustrated in Figure 8. The hub genes with the highest number of directed edges at the network of C1 and T2 were *HCEF1* (fructose-1,6-bisphosphatase, VIT_08s0007g01570), including 34 edges, and *BZIP9* (basic leucine zipper 9, VIT_04s0008g02750) including 18 edges, respectively (Figure 8). The highly correlated module at the T1 stage was the gray module. The genes that are not belonging to other modules were enriched into a gray module, so gray modules were not real module. Therefore, the modules of the T1 stage were not analyzed. The networks of C2, C4, C5, T3, and T4 were shown in Supplemental Figure S2. Furthermore, the functional annotation of the hub genes at the C2, C4, C5, T3, and T4 stages were as late embryogenesis abundant protein; inactive protein kinase; hypothetical protein; alpha beta-hydrolase-like protein; pentatricopeptide repeat-containing protein; microtubule-binding protein and putative invertase inhibitor; and sodium/hydrogen exchanger 2; respectively.

### 3.6. qRT-PCR Assay

**Figure 9:**
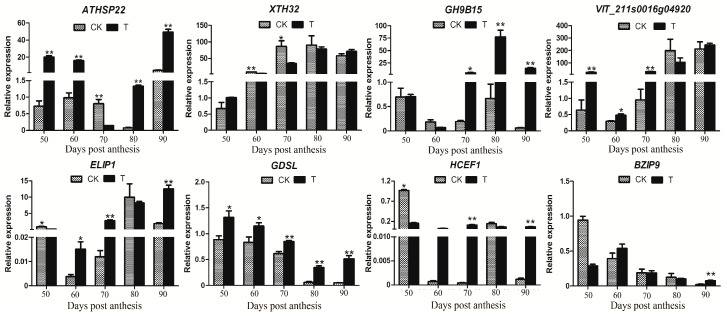
qRT-PCR expression patterns of both six DEGs and two hub genes detected in the RNA-Seq profiles of the control and the treatment. The X-axis represents the berry development stages; the Y-axis represents the relative level of expression. Three biological replicates are used for qRT-PCR validation. Significant differences between means were made using ANOVA. The asterisk (*) stands for the levels of significant difference (*p* value ≤ 0.05).

To further verify the reliability of the RNA-Seq data, six differentially expressed genes (including *ATHSP22*, *XTH32*, *VIT_211s0016g04920, GH9B15*, *GDSL,* and *ELIP1*) and two hub genes (*BZIP9* and *HCEF1*) were selected for real-time quantitative reverse transcription (qRT)-PCR analysis. The qRT-PCR results indicated that the expression levels of *GH9B15, ELIP1, HCEF1,* and *VIT_211s0016g04920* genes after the treatment of riboflavin were significantly higher than the control at 70 dpa (Figure 9). However, the expression levels of *ATHSP22* and *XTH32* genes were significantly down-regulated in the treatment at 70 dpa. Compared with the control, the *GDSL* gene was significant differentially expressed after riboflavin treatment at all the berry development stages. In addition, the melt curve and amplification efficiency details of eight genes were shown in Supplemental Figure S3. The results from transcriptome data and the one obtained by qRT-PCR were largely consistent.

## 4. Discussion

ROS functioned as a signaling molecule during the fruit-ripening process [34]. The previous study showed that the treatment of riboflavin promoted the ripening of ‘Kyoho’ berries 16 d earlier than the control [18]. However, the effect of riboflavin treatment on the disturbance of gene expressions of ‘Kyoho’ is still unclear. Transcriptomic analysis could provide the clues to dissect the molecular mechanisms of plant growth and development. Therefore, the RNA-seq was further employed to understand the gene expressions after riboflavin treatment in this study.

There are not any common DEGs obtained from the comparisons of the treatment and the control. It is possible due to the few DEGs identified at the early berry development stage. Based on the results of TCseq (Figure 2), most of the genes have similar expression patterns. That is to say, the treatment of riboflavin didn’t result in any great fluctuation of the gene expressions. It just had an effect on some key pathways. Accordingly, the enriched analysis of TCseq, DEGs, and WGCNA analysis all indicated that some common pathways were associated with chlorophyll binding (Figure 3A and Figure 5A), photosynthesis (Figure 3B and Figure 7), pigment binding (Figure 5A), and photosynthesis–antenna proteins (Figure 5B and Figure 7) in both GO and KEGG enrichment analysis.

Chloroplasts play a key role in plant growth and development by housing the photosynthesis machinery [35]. Photosynthesis plays a major role in the process of fruit development, mainly including the light reaction, photorespiration, and Calvin cycle processes; while photorespiration and the Calvin cycle process more occurred in young fruit stages [36]. Plants convert light energy into chemical energy to provide energy to organs by photosynthesis [37]. The decrease in the protein content associated with photosynthesis was observed during the development of grape berries, especially after veraison [38,39]. In this study, the pathways of the photosynthesis (Figure 7) and photosynthesis–antenna proteins (Figure 5B) were indeed present in the C1 and C3–T3 stages.

Early light-induced proteins (ELIP) were thylakoid proteins that were transiently induced by light [40]. ELIPs belong to the family of chlorophyll a/b binding proteins [41], and were predicted to bind chlorophyll and function in photoprotection under high light and other abiotic stresses [42]. The overexpression of *ELIP* genes in *Arabidopsis* suggested that the ELIP protein reduced the chlorophyll content by inhibiting chlorophyll biosynthesis [43]. A lack of *ELIPs* was strongly susceptible to photooxidative stress in *Arabidopsis* [44]. The qRT-PCR results showed that the expression level of *ELIP1* was significant higher in the treatment than the control at 70 dpa (Figure 9). The previous results indicated that riboflavin treatment reduced the content of chlorophyll, but promoted the content of anthocyanin [18]. In addition, the gene co-expression network analysis identified the *HCEF1* gene as a hub node, which functioned as fructose-1,6-bisphosphatase. Previous reports indicated that the fructose-l,6-bisphosphatase (FBPase) played a crucial role in photosynthesis in potato [45].

Fatty acids are the building blocks for the majority of cellular lipids, which are not only essential for the function of the membrane, but also are necessary for fruit growth and development [46,47]. The expression of the genes in Cluster 5 from the TCseq results were obviously up-regulated in the treatment; the enrichment analysis revealed that the pathway of fatty acid metabolism was enriched in this cluster (Figure 3B). Previous research had shown that the GDSL proteins were somehow involved in fatty acids degradation in seeds of *Arabidopsis* [48]. In higher plants, GDSL lipase was a member of the lipase superfamily [49]. Previous studies had reported that the GDSL family from *Arabidopsis thaliana* consisted of 108 members [50]. Meanwhile, *Vitis vinifera*, *Populus trichocarpa*, *Sorghum bicolour*, and *Physcomitrella patens* contained 96 members, 126 members, 130 members, and 57 members, respectively [51]. The GDSL lipases were found as multifunctional enzymes, which were associated with many physiological processes, including plant growth and development, morphogenesis, lipid metabolism, stress resistance, and the formation of tissue and organs in plants [52,53,54].

The primary cell walls and secondary cell walls are major components of cell walls. However, the secondary cell walls are mainly composed of xylan, cellulose, and hemicelluloses. The acetylation contributes to the formation of secondary wall architecture [55]. The GDSL esterase was an acetyl xylan esterase, which removed acetyl groups from the xylan backbone [55]; i.e., the GDSL lipase was from the deacetylation of the xylan backbone. Then, it promoted the cell wall softening. The GDSL esterase/lipase was one of the DEGs identified in this study. The qRT-PCR showed that the expression of *GDSL* (VIT_05s0020g04840) was significantly higher in the treatment than in the control (Figure 9). The results indicated that the riboflavin treatment induced the *GDSL* gene expression, and then promoted the berries development and cell wall softening of ‘Kyoho’ berries.

Fruit softening was correlated with the structure and composition of the cell wall [56], which coordinated a range of interdependent actions of hydrolytic enzymes, including xyloglucan endotransglucosylase/hydrolase (XTH), β-galactosidase, and pectin methylesterase, and so on [57]. XTH enzymes play a major role in fruit softening through loosening the cell wall by the disassembly of xyloglucan [56]. Moreover, the endoglucanases and polygalacturonases were involved in fruit softening [58]. The accumulation of *FaEGase1* (encoding a putative endoglucanase) mRNA was strongly associated with fruit ripening in strawberry [59]. In this study, the annotation of *GH9B15* was associated with endoglucanase. RNA-seq showed that *XTH32* and *GH9B15* genes were significantly down-regulated and up-regulated in the treatment group, respectively (Table 2). The qRT-PCR showed that the expression level of *XTH32* (VIT_06s0061g00550) was indeed higher in the control than the treatment and had a significant difference at 70 dpa (Figure 9). However, the expression level of *GH9B15* (VIT_02s0025g00430) was higher in the treatment than the control and had a significant difference at 70 dpa to 90 dpa (Figure 9). The results indicated that the riboflavin treatment promoted the fruit softening by regulating the expression of the *XTH32* and *GH9B15* genes.

Heat shock proteins (HSPs) are a superfamily of molecular chaperone proteins and stress proteins that accumulate under heat shock conditions as well as under other abiotic and biotic stresses [60,61]. In addition, it could hold unfolded protein back aggregate, which protects plants from cellular protein malfunction and stress [61]. The HSPs were classified into different families according to their molecular weight, amino acid sequence homology, and function, including the Hsp100 family, Hsp90 family, Hsp70 family, Hsp60 family, and small HSP (sHSPs) family [62]. *ATHSP22* was screened as a DEG in this study, and belonged to the small HSP family. Some studies have reported that small sHSPs play a key role in tolerance to abiotic stresses including heat, cold, drought, salinity, and oxidative stress [63].

The sHSPs can lead to oxidative damage, and the accumulation of reactive oxygen species caused the oxidative damage [64,65]. The gene expression of *HSPs* was not only regulated by environmental stress, but also by plant developmental signals [66,67]. For example, the *sHSP21* played a major role in photosystem II against oxidative stress and regulates pigment development in tomato [68]. The qRT-PCR showed that the expression level of *ATHSP22* (VIT_18s0089g01270) was higher in the treatment than the control, and had a significant difference, except for 70 dpa (Figure 9). The riboflavin treatment on ‘Kyoho’ berries lead to the ROS accumulation at an early stage; the sHSPs are able to function synergistically as ROS scavengers with cell antioxidant systems [69]. Therefore, the *ATHSP22* gene and the antioxidant system coordinated to regulate the ROS balance and promote the early ripening of grape berries.

## 5. Conclusions

RNA-seq was carried out to identify the molecular mechanism underlying the development of the ‘Kyoho’ berry after riboflavin treatment. In this study, the analysis of DEGs indicated that riboflavin treatment triggered the up-regulation of some genes (*GDSL*, *GH9B15,* and *ELIP1*) and the down-regulation of others (*XTH32* and *ATHSP22*). In addition, TCseq and WGCNA analysis revealed that some pathways were enriched and associated with photosynthesis. As a result, the ripening-related genes, including the photosynthesis-related *ELIP1* (VIT_05s0020g04110), growth and development-related *GDSL* (VIT_05s0020g04840), oxidative stress-related *ATHSP22* (VIT_18s0089g01270), berry softening-related *XTH32* (VIT_06s0061g00550) and *GH9B15* (VIT_02s0025g00430) were affected by riboflavin treatment. The transcription analysis of the riboflavin treatment indicated that early ripening of the ‘Kyoho’ berry was related to the changes of the expression level of these genes.

## Figures and Tables

**Table 1:** Riboflavin treatment and sampling time points.

Days Post-Anthesis (DPA)					
Control sampling time	50 (C1)	60 (C2)	70 (C3)	80 (C4)	90 (C5)
Treatment sampling time	50 (T1)	60 (T2)	70 (T3)	80 (T4)	90 (T5)

Both the sampling and treatment were performed in 2016.

**Table 2:** List of differentially expressed genes for the treatment over the control after riboflavin treatment. FDR: false discovery rate.

Stages	Genes ID	Genes Name	Regulated	FDR	Log2FoldChange	Annotation Function
**C1 vs. T1**	VIT_08s0007g04250	*VIT_208s0007g04250*	up	3.24 × 10^−68^	Inf	Probable glycosyltransferase
	VIT_05s0020g04840	*GDSL*	up	9.92 × 10^−65^	2.769179	GDSL esterase/lipase
	VIT_04s0044g01160	*VIT_204s0044g01160*	up	1.29 × 10^−55^	Inf	Probable cysteine desulfurase-like
	VIT_02s0025g04950	*VIT_202s0025g04950*	up	2.15 × 10^−44^	Inf	Hypothetical protein
	VIT_16s0039g02140	*PRK*	up	3.39 × 10^−27^	Inf	Phosphoribulokinase, chloroplastic
	VIT_16s0013g00870	*PEI1*	up	3.59 × 10^−24^	Inf	Zinc finger CCCH domain-containing protein
	VIT_13s0067g03890	*KCS19*	down	0.0069	−1.36784	3-ketoacyl-CoA synthase 21
**C2 vs. T2**	VIT_04s0008g03940	*RD22*	up	0.000148	2.746652	Dehydration-responsive protein RD22 (Precursor)
	VIT_07s0005g01790	*LACS1*	up	1.07 × 10^−9^	2.043324	Long chain acyl-CoA synthetase 1
	VIT_06s0061g00550	*XTH32*	down	4.39 × 10^−49^	−2.0868	Probable xyloglucan endotransglucosylase/hydrolase Protein 32 (Precursor)
	VIT_06s0004g02560	*VIT_206s0004g02560*	down	1.52 × 10^−23^	−1.45724	Ripening-related protein grip22 (Precursor)
	VIT_16s0022g00960	*VIT_216s0022g00960*	down	2.53 × 10^−23^	−1.49248	21 kDa protein (Precursor)
	VIT_08s0007g08330	*ADPG1 PGAZAT*	down	1.05 × 10^−10^	−1.87416	Polygalacturonase (Precursor)
**C3 vs. T3**	VIT_11s0016g04920	*VIT_211s0016g04920*	up	1.95 × 10^−215^	4.90208	Early nodulin-93 OS=Glycine max (Soybean)
	VIT_02s0025g00430	*GH9B15*	up	2.58 × 10^−33^	6.101505	Endoglucanase (Precursor)
	VIT_05s0020g04110	*ELIP1*	up	1.86 × 10^−8^	3.826895	Early light-induced protein 1
	VIT_18s0089g01270	*ATHSP22*	down	3.22 × 10^−31^	−3.35075	22.7 kDa class IV heat shock protein (Precursor)
	VIT_02s0154g00320	*VIT_202s0154g00320*	down	3.95 × 10^−27^	−3.06725	14 kDa proline-rich protein DC2.15 (Precursor)
	VIT_12s0028g02830	*VIT_212s0028g02830*	down	2.45 × 10^−27^	−2.89329	Trans-resveratrol di-O-methyltransferase
	VIT_12s0028g01880	*VIT_212s0028g01880*	down	5.60 × 10^−25^	−2.71443	Trans-resveratrol di-O-methyltransferase

## Supplementary Materials

The following are available online at https://www.mdpi.com/2073-4425/10/7/514/s1. Supplemental Table S1. Primers used for quantitative PCR analysis in this study; Supplemental Table S2. Overview of the ‘Kyoho’ transcriptome sequencing data; Supplemental Figure S1. The optimization of partitions of clusters in TCseq analysis; Supplemental Figure S2. Cytoscape representation of co-expressed genes with edge weight ≥0.1. The important hub gene was noted with yellow. (A) The hub gene of C2 stage (B) The hub gene of C4 stage (C) The hub gene of C5 stage (D) The hub gene of T3 stage (E) The hub gene of T4 stage, respectively; Supplemental Figure S3. The amplification efficiency and melt curve details of eight genes in qRT-PCR analysis. (A) The melt curve of *VIT_211s0016g04920*, *ATHSP22* and *Ubiquitin* genes; (B) The amplification efficiency details of *VIT_211s0016g04920*, *ATHSP22* and *Ubiquitin* genes; (C) The melt curve of *XTH32*, *ELIP1* and *Ubiquitin* genes; (D) The amplification efficiency details of *XTH32*, *ELIP1* and *Ubiquitin* genes; (E) The melt curve of *GDSL*, *BZIP9* and *Ubiquitin* genes; (F) The amplification efficiency details of *GDSL*, *BZIP9* and *Ubiquitin* genes; (G) The melt curve of *HCEF1*, *GH9B15* and *Ubiquitin* genes; (H) The amplification efficiency details of *HCEF1*, *GH9B15* and *Ubiquitin* genes, respectively.

## Abbreviations

ROS	Reactive oxygen species
SOD	Superoxidase dismutase
PCA	Principal components analysis
GO	Gene Ontology
KEGG	Kyoto Encyclopedia of Genes and Genomes
DEGs	Differentially expressed genes
WGCNA	Weighted gene co-expression network analysis
HCEF1	Fructose-1,6-bisphosphatase
BZIP9	Basic leucine zipper 9
ELIP	Early light induced proteins
XTH	Xyloglucan endotransglucosylase/hydrolase
HSPs	Heat shock protein
