# Supplementary material for: Transcriptional Analysis of the Early Ripening of ‘Kyoho’ Grape in Response to the Treatment of Riboflavin
Source: Genes (Basel). 2019 Jul 6;10(7):514. doi: 10.3390/genes10070514 (PMC6678464; doi:10.3390/genes10070514)
Supplement: Supplementary file 1 [file genes-10-00514-s001.pdf]

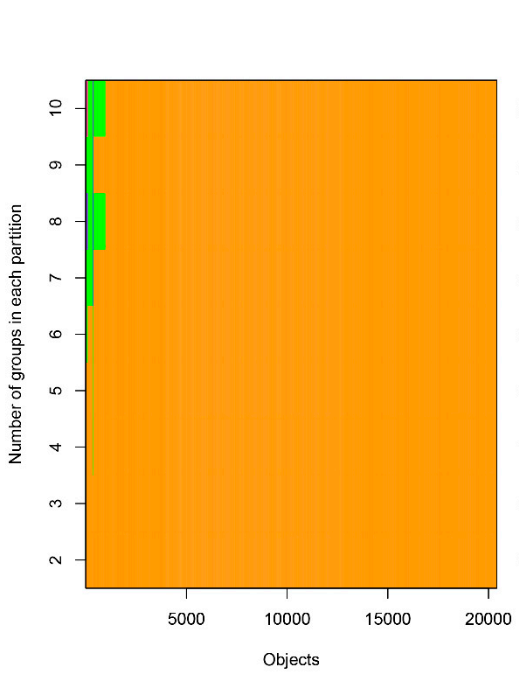

Supplemental Figure S1. The optimization of partitions of clusters in TCseq analysis.

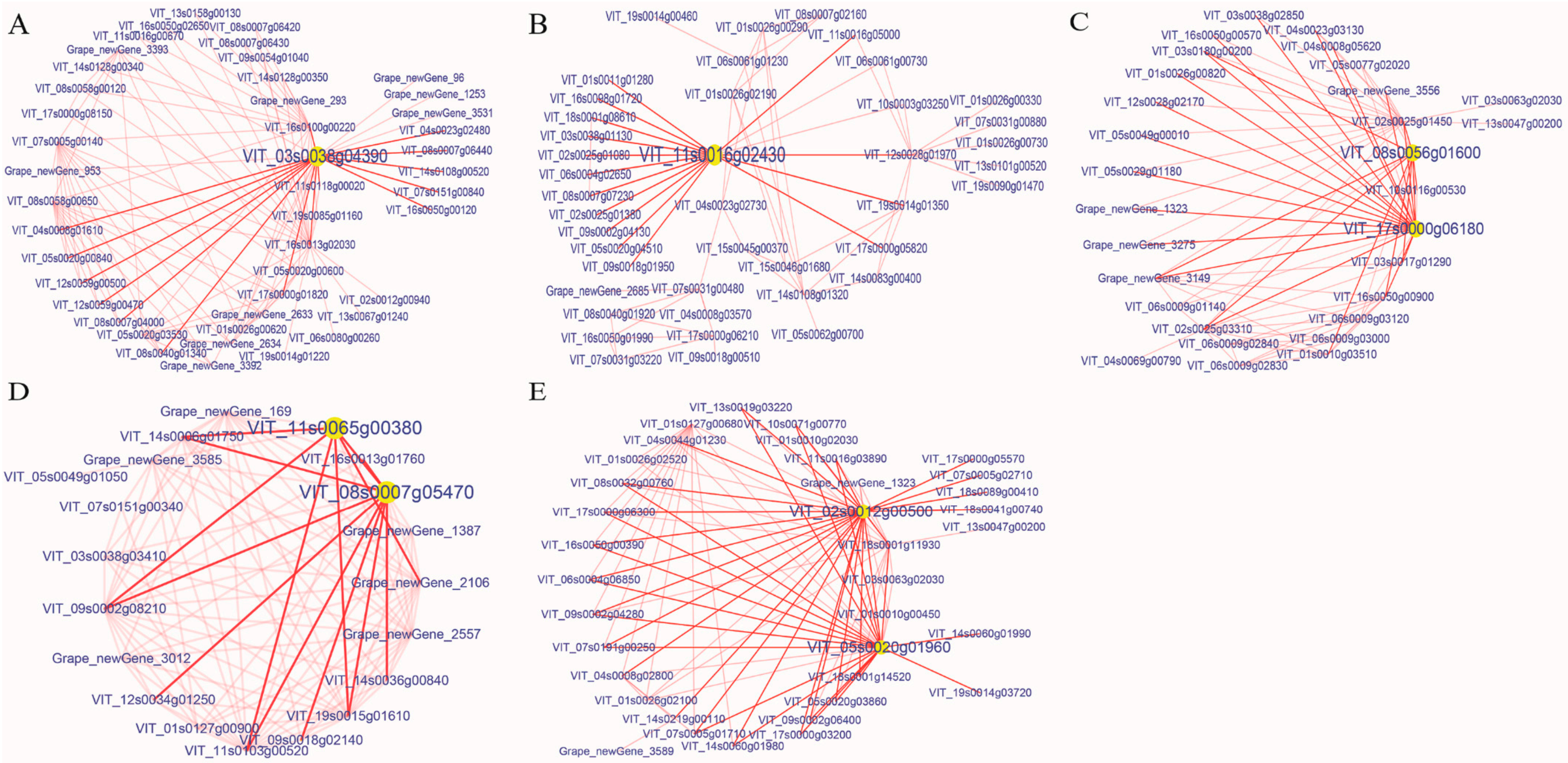

Supplemental Figure S2. Cytoscape representation of co-expressed genes with edge weight ≥0.1. The important hub gene was noted in yellow. (**A**) The hub gene of the C2 stage; (**B**) The hub gene of the C4 stage; (**C**) The hub gene of the C5 stage; (**D**) The hub gene of the T3 stage; and (**E**) The hub gene of T4 stage, respectively.

Supplemental Table S1. Primers used for quantitative PCR analysis in this study.

Gene name	Gene ID		Sequence (5'–3')
ELIP1	VIT_05s0020g04110	Forward	TCACAGTCACGTCAACCGAT
		Reverse	CCGCAACAAAGCCTACCAT
XTH32	VIT_06s0061g00550	Forward	AGGTTACTTCAGTGCTGCCATC
		Reverse	GAGGCTTATCCAGTGTTGTTCC
VIT_211s0016g04920	VIT_11s0016g04920	Forward	TGGCAAAGAATGTGGCTGG
		Reverse	GGCAATGCTGGCAACAACT
ATHSP22	VIT_18s0089g01270	Forward	TCTTTGCCCAACTTGTCGTC
		Reverse	GCCTGTTTGGAGGTTTCACTT
GH9B15	VIT_02s0025g00430	Forward	AAGACCGACGCAGACTCATT
		Reverse	CACATTGGACTCCTCCGATT
BZIP9	VIT_04s0008g02750	Forward	GTGGTGATGGCGAAGGGTAT
		Reverse	TGAGCGGAGGTTTCTTAGGG
HCEF1	VIT_08s0007g01570	Forward	GGCCGTGGAAGAGAGTTACT
		Reverse	CGCACCTTTGTTCTGTTGAG
GDSL	VIT_05s0020g04840	Forward	TGGAGTGGTCTGTGATCCTT
		Reverse	CTCATTCACCCTCACTGCTC

Supplemental Table S2. Overview of the 'Kyoho' transcriptome sequencing.

Sampl es	Clean reads	Clean bases	GC Content (%)	Q20 (%)	Q30 (%)	Unique Map	Mapping rate (%)
C11	35,278,395 (98.58%)	10,523,043,944	47.14	97.32	94.47	46,381,190	65.74
C12	27,486,349 (98.42%)	8,192,198,634	47.40	97.37	94.48	34,840,243	63.38
C21	30,867,813 (98.43%)	9,199,338,978	47.16	97.47	94.63	40,546,408	65.68
C22	28,184,064 (98.43%)	8,399,870,658	46.86	97.47	94.68	38,417,757	68.16
C31	25,718,449 (98.45%)	7,667,592,892	48.00	97.13	94.09	32,236,962	62.67
C32	31,863,349 (98.46%)	9,501,218,124	48.25	97.15	94.14	39,693,382	62.29
C41	28,700,254 (98.50%)	8,555,888,188	47.75	97.23	94.26	36,753,426	64.03
C42	27,709,439 (98.41%)	8,260,755,972	47.21	97.40	94.63	36,964,844	66.70
C51	33,098,799 (98.56%)	9,859,370,600	46.56	96.83	92.02	44,398,084	67.07
C52	32,482,251 (98.62%)	9,678,549,430	46.99	96.91	92.16	44,012,511	67.75
T11	35,990,802 (98.49%)	10,731,368,632	47.42	97.40	94.60	47,470,731	65.95
T12	36,180,711 (98.53%)	10,782,502,072	47.83	97.44	94.61	47,826,003	66.09
T21	26,892,431 (98.61%)	8,019,430,484	46.82	97.51	94.72	36,084,511	67.09
T22	24,445,704 (98.25%)	7,287,194,396	47.18	97.38	94.47	32,048,688	65.55
T31	25,908,751 (98.37%)	7,721,128,896	47.83	97.27	94.35	33,218,794	64.11
T32	32,503,991 (98.42%)	9,684,925,524	47.40	97.38	94.52	43,022,785	66.18
T41	26,882,981 (98.28%)	8,015,950,740	47.60	97.19	94.20	34,737,161	64.61
T42	26,466,916 (98.25%)	7,889,034,102	47.82	97.22	94.21	34,236,505	64.68
T51	28,840,052 (98.04%)	8,591,264,612	46.72	97.08	94.02	39,519,828	68.52
T52	27,216,227 (98.17%)	8,111,370,698	47.10	97.17	94.13	36,778,933	67.57

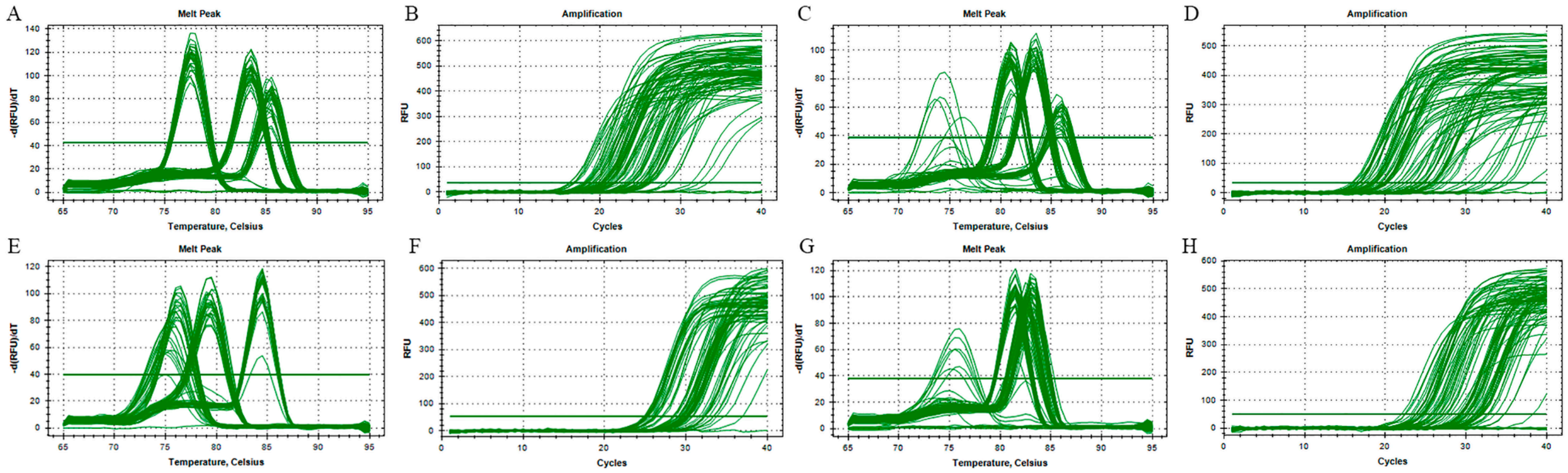

Supplemental Figure S3. The amplification efficiency and melt curve details of eight genes in qRT-PCR analysis. (**A**) The melt curve of *VIT_211s0016g04920*, *ATHSP22*, and *Ubiquitin* genes; (**B**) The amplification efficiency details of *VIT_211s0016g04920*, *ATHSP22*, and *Ubiquitin* genes; (**C**) The melt curve of *XTH32*, *ELIP1*, and *Ubiquitin* genes; (**D**) The amplification efficiency details of *XTH32*, *ELIP1*, and *Ubiquitin* genes; (**E**) The melt curve of *GDSL*, *BZIP9*, and *Ubiquitin* genes; (**F**) The amplification efficiency details of *GDSL*, *BZIP9*, and *Ubiquitin* genes; (**G**) The melt curve of *HCEF1*, *GH9B15*, and *Ubiquitin* genes; (**H**) The amplification efficiency details of *HCEF1*, *GH9B15*, and *Ubiquitin* genes, respectively.
